# Anagenetic speciation in Ullung Island, Korea: genetic diversity and structure in the island endemic species, *Acer takesimense* (Sapindaceae)

**DOI:** 10.1007/s10265-012-0529-z

**Published:** 2012-10-23

**Authors:** Koji Takayama, Byung-Yun Sun, Tod F. Stuessy

**Affiliations:** 1 Department of Systematic and Evolutionary Botany, Biodiversity Center, University of Vienna, Rennweg 14, 1030 Vienna, Austria; 2 Faculty of Biological Sciences, College of Natural Sciences, Chonbuk National University, Chonju, South Korea

**Keywords:** Anagenetic speciation, Cladogenesis, Endemic plants, Korean Peninsula, Oceanic island, Phyletic speciation

## Abstract

Anagenetic speciation is an important mode of speciation in oceanic islands; one-fourth of the endemic plants are estimated to have been derived via this process. Few studies, however, have critically examined the genetic consequences of anagenesis in comparison with cladogenesis (involved with adaptive radiation). We hypothesize that endemic species originating via anagenetic speciation in a relatively uniform environment should accumulate genetic variation with limited populational differentiation. We undertook a population genetic analysis using nine nuclear microsatellite loci of *Acer takesimense*, an anagenetically derived species endemic to Ullung Island, Korea, and its continental progenitor *A. pseudosieboldianum* on the Korean Peninsula. Microsatellite data reveal a clear genetic distinction between the two species. A high *F* value in the cluster of *A. takesimense* was found by Bayesian clustering analysis, suggesting a strong episode of genetic drift during colonization and speciation. In comparison with *A. pseudosieboldianum*, *A. takesimense* has slightly lower genetic diversity and possesses less than half the number of private and rare alleles. Consistent with predictions, weak geographical genetic structure within the island was found in *A. takesimense*. These results imply that anagenetic speciation leads to a different pattern of specific and genetic diversity than often seen with cladogenesis.

## Introduction

Oceanic islands have long been recognized as fascinating natural laboratories for the study of evolution (Bramwell and Caujapé-Castells 2011; Carlquist 1974; Darwin 1842; Grant 1996; MacArthur and Wilson 1967; Wallace 1881; Whittaker 1998). Due to being relatively small land masses, geographically isolated, with known geological ages, and relatively simple biota with high levels of endemism, oceanic islands provide great opportunities for investigating evolutionary processes of organisms, especially in contrast to more complex continental situations.

The most commonly described evolutionary process in oceanic islands has emphasized “cladogenesis” involved with adaptive radiation. During cladogenesis, an initial founder population divides into several lineages through isolation and subsequent adaptation to markedly different ecological zones, bringing about different morphological or physiological traits (Carlquist 1974; Futuyma 1997; Rundell and Price 2009; Schluter 2001), sometimes of a dramatic nature as seen, for instance, in the silverswords (Asteraceae) of the Hawaiian Islands (Baldwin 1997; Baldwin and Wessa 2000) or *Echium* (Boraginaceae) of the Canary Islands (Böhle et al. 1996; Marrero-Gómez et al. 2000). Morphologically or physiologically diverging populations accumulate some genetic differences, but the more conspicuous pattern is partitioning of the gene pool into restricted genetic lines (Schluter 1996).

Another speciation process common in oceanic islands is anagenetic speciation (also known as simple geographical or phyletic speciation). During anagenetic speciation, an initial founder lineage simply transforms genetically and morphologically through time without further specific differentiation (Stuessy et al. 1990; Stuessy et al. 2006; Stuessy 2007; Whittaker et al. 2008). This speciation process is important in plant evolution on oceanic islands, with levels of endemic specific diversity explainable by this process ranging from 7 to 88 %, with a mean for all islands of 25 % world-wide (as estimated by floristic surveys; Stuessy et al. 2006).

It has been hypothesized that the two major modes of speciation in oceanic islands result not only in distinct levels of species diversity but also in different levels of genetic diversity within endemic species (Stuessy 2007). With both cladogenetic and anagenetic speciation, the founder populations to the island bring only a small portion of the genetic variation originally contained in the usually larger and more broadly distributed continental progenitors (Frankham 1997). During cladogenesis, the depauperate gene pool of the founding populations becomes partitioned and channeled under intense selection into different habitats, leading to noticeable morphological and genetic differences among populations. These distinct populations are worthy of being called different species, but each contains a low level of genetic variation. During anagenesis, on the other hand, the initial founder populations accumulate genetic variation through time within a relatively uniform environment without any splitting event. Over many generations, sufficient genetic and morphological divergence accrues so that recognition of a new species is warranted. Molecular studies so far completed have revealed higher or only slightly lower levels of genetic variation in two anagenetically derived species, *Dystaenia takesimana* (Pfosser et al. 2005) and *Acer okamotoanum* (Takayama et al. 2012) in Ullung Island, off the coast of Korea, in comparison to their continental progenitors. The endemic species also show no geographical partitioning of the genetic variation within the island. More studies are needed, however, to synthesize more data and examine more critically the genetic consequences of anagenetic speciation in comparison with those from the classical cladogenetic model.

Ullung Island is an ideal setting for investigating the genetic consequences of anagenetic speciation because it consists of only a single island, and it contains the highest levels of anagenetic speciation so far recorded among oceanic islands (Stuessy et al. 2006). Ullung Island is located 137 km east of the Korean Peninsula, extending from 37°27′ to 37°33′N and 130°47′ to 130°56′E; the total area is 73 km^2^ and the highest peak is 984 m. It is of volcanic origin, with no known connections to the peninsula, and with a geological age of approximately 1.8 million years (Kim 1985). The flora contains approximately 700 vascular plant species including 500 natives of which 37 angiosperms are endemic (Lee and Yang 1981). One of the important characteristics of the endemics is that most of them are single representatives of different genera that appear to have diverged via simple anagenetic change from continental progenitors (Sun and Stuessy 1998). The relationships between progenitors in continental areas and endemic species in Ullung Island have been confirmed in several genera by phylogenetic studies (Oh et al. 2010; Pfosser et al. 2002, 2005, 2011; Yang and Pak 2006; Yang et al. 2012), indicating that most of the progenitor species are distributed in the Korean peninsula and/or Japanese Archipelago.

Two endemic *Acer* (Sapindaceae) species, *A. okamotoanum* Nakai and *A. takesimense* Nakai, occur on Ullung Island. Morphological and phylogenetic studies have shown that these two endemics are not closely related to each other but rather have had independent anagenetic origins from different continental progenitors, *A. mono* Maxim. and *A. pseudosieboldianum* Kom., respectively (Ackerly and Donoghue 1998; Pfosser et al. 2002; Sun and Stuessy 1998). Crawford (2010) listed these two species pairs as good examples of progenitor-derivative speciation, which would provide appropriate systems for studying plant speciation. This present paper focuses on the genetic consequences of anagenetic speciation in one of these endemic *Acer* species, *A. takesimense*.

*Acer takesimense* is an endemic tree widely distributed in Ullung Island (Yim et al. 1981). Its putative progenitor, *A. pseudosieboldianum*, is found in the cool-temperate deciduous forests of northeastern Asia, Manchuria, Ussuri River, China, and Korea (van Gelderen et al. 1994). Nakai (1918) stated that the former species is closely related to the latter species, and van Gelderen et al. (1994) treated *A. takesimense* as a subspecies of *A. pseudosieboldianum*. The species relationships in section *Palmata* that contain both species are still unclear. Although it is possible to consider that *A. takesimense* might have derived from other *Acer* species in section Palmata, recent molecular work combined with chloroplast DNA, ITS and AFLP data support that the most probable ancestor of *A. takesimense* was *A. pseudosieboldianum* from the Korean Peninsula (Pfosser et al. 2002). The AFLP studies also have indicated that the two species are genetically distinguishable.

In this study we investigate genetic consequences of anagenetic speciation of *A. takesimense* from *A. pseudosieboldianum* by examining patterns of genetic diversity and structure in populations using nuclear microsatellite markers. We address three main questions: (1) Is there clear genetic differentiation between the two species? (2) How much genetic diversity exists within island populations of *A. takesimense* in comparison to continental populations of *A. pseudosieboldianum*? (3) Is there any geographic structuring of genetic variation among populations of *A. takesimense* within the island?

## Materials and methods

### Plant material

**Fig. 1:**
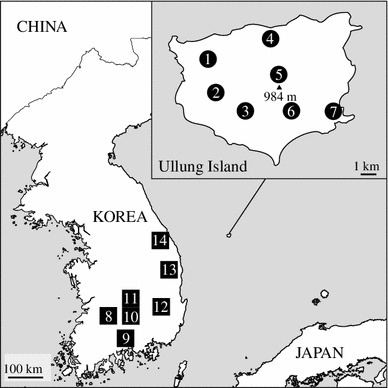
Location of Ullung Island and populations of *Acer takesimense* (*1*–*7*) and *A. pseudosieboldianum* (*8*–*14*) analyzed in this study

We collected leaf samples from 130 individuals of *Acer takesimense* from seven populations in Ullung Island, and 133 individuals of *A. pseudosieboldianum* from seven populations in the Korean Peninsula (Table 1; Fig. 1). The leaf samples were desiccated with silica gel in zip-lock plastic bags until use in the laboratory. Voucher specimens of these samples were deposited in JNU and WU.

**Table 1:** Populations of *Acer takesimense* from Ullung Island and *A. pseudosieboldianum* from the Korean Peninsula analyzed for genetic diversity with nuclear microsatellites

Taxon	Population	Locality	Altitude (m)	Voucher	*N*_POP_	*N*
*Acer takesimense*	1	Ullung Island, Taeha-ri-Jung-ri	120	TS17551	ca. 50	20
	2	Ullung Island, Namseo-ri	280	TS17589	>100	18
	3	Ullung Island, Namyang-ri	120	TS17603	>100	20
	4	Ullung Island, Chusan to Nari	220	SUN4117	>100	18
	5	Ullung Island, Seongin Mt.	900	SUN4145	ca. 30	16
	6	Ullung Island, Sadong-ri	300	TS17617	>100	20
	7	Ullung Island, Do-dong National Forest	140	SUN4006	ca. 50	18
	Total					130
*A. pseudosieboldianum*	8	Prov. Chonbuk, Moak Mt.	405	TS16007	ca. 100	17
	9	Prov. Chonbuk, Chiri Mt.	1,100	TS16020	>100	20
	10	Prov. Chonbuk, Juksang Mt.	850	TS16024	ca. 100	20
	11	Prov. Chonbuk, Juksang Mt.	980	TS16030	>100	20
	12	Prov. Kyungnam, Palgong Mt.	740	TS17637	>1,000	17
	13	Prov. Kyungbuk, Irwol Mt.	820	TS17632	ca. 100	20
	14	Prov. Kangwon, Odae Mt.	930	TS16072	>1,000	19
	Total					133

*TS* Tod F. Stuessy, *SUN* Byung-Yun Sun, *N*_*POP*_ the estimated number of individuals in population by field observation, *N* the total number of analyzed samples

### DNA extraction and microsatellite genotyping

Total genomic DNA was extracted from dried leaves using the DNeasy 96 Plant Kit (Qiagen, Hilden, Germany). We selected ten microsatellite markers, which were isolated from *A. pseudosieboldianum* according to the repeatability and scoring convenience of the markers (Takayama et al. 2011). For PCR amplification, we applied the 5′-tailed primer method (Boutin-Ganache et al. 2001) to label amplified fragments for detection in the capillary sequencer, which was done in the same way as in the previous study (Takayama et al. 2011). Four combinations of multiplex PCR amplification were performed using a slightly modified protocol of the Qiagen Multiplex PCR Kit (Qiagen, Hilden, Germany). The multiplex combinations were as follows: A7DU1, A82LK, A88LE with 6-FAM, BDG7D, A08Z1, AY8V2 with VIC, APT6F, ASQGF with NED, AVM1G, AW0F6 with PET. A multiplex PCR amplification was performed in a final volume of 3 μL with 0.2 μM of each reverse primer, 0.04 μM of each forward primer, and 0.4–0.6 μM of fluorescent dye-labeled primer (0.6 μM for 6-FAM and VIC, 0.4 μM for NED and PET). Touchdown thermal cycling programs were used as follows: initial denaturation at 95 °C for 15 min, followed by 25 cycles of denaturation at 95 °C for 30 s, annealing at 63 °C for 90 s (decreased 0.5 °C per cycle), and extension at 72 °C for 60 s; and by 20 cycles of denaturation at 95 °C for 30 s, annealing at 53 °C for 90 s, and extension at 72 °C for 60 s; a final extension step was performed at 60 °C for 30 min. For genotyping, 1.0 μL of 25–40 times diluted PCR product mix was mixed with 10 μL of HiDi formamide (Applied Biosystems, Warrington, UK) and 0.1 μL of GeneScan600 LIZ size standard (Applied Biosystems) and run on an automated sequencer (ABI 3130xl). Scoring of fluorescence peaks was performed using GeneMarker (SoftGenetics LLC).

### Data analysis

The statistical significance of deviation from Hardy–Weinberg equilibrium (HWE), and linkage disequilibrium between loci in each population, were conducted with the Markov chain method (10,000 dememorisation steps, 1,000 batches, 500 iterations per batch) using GENEPOP 4.0 (Raymond and Rousset 1995). Null allele frequency was calculated following Brookfield (1996) using Micro-Checker 2.2.3 (van Oosterhout et al. 2004).

Genetic diversity, in terms of allelic richness (*A*_R_), observed proportion of heterozygotes (*H*_O_), expected proportion of heterozygotes (*H*_E_), and total number of alleles, was evaluated for each species and population using FSTAT 2.9.3.2 (Goudet 1995). Allelic richness was standardized for 16 individuals based on minimum sample size of populations using the rarefaction method (Hurlbert 1971). The inbreeding coefficient (*F*_IS_) was calculated in the program. We also counted the number of private alleles (unique to one group), and rare alleles (defined as alleles with a frequency <10 % in the total group) per individuals in each group. Three categories were constructed for these estimates: (i) each species is treated as a group; (ii) the seven populations of *A. takesimense* as a group and the seven populations of *A. pseudosieboldianum* as seven different groups; and (iii) each population in both species as a group.

Genetic variation between taxa, among and within populations was evaluated by AMOVA using ARLEQUIN 3.5.1.2 (Excoffier et al. 2005). The AMOVA analyses were done by three categories: (a) all samples from the two species, (b) *A. takesimense*, and (c) *A. pseudosieboldianum*. Statistical significance of the variance components was tested by calculating their probabilities based on 1,023 permutations. Genetic distance among populations was calculated by *D*_A_ genetic distance (Nei et al. 1983), and a neighbour-joining tree was reconstructed based on the distance using Populations 1.2.30 (Langella 1999). Statistical significance of the best topology was estimated with 1,000 bootstrap replicates. The Bayesian clustering method (Falush et al. 2003; Pritchard et al. 2000) implemented in STRUCTURE 2.3.3 (Falush et al. 2007; Hubisz et al. 2009; Pritchard et al. 2000) was used for evaluation of genetic structure as well. We conducted STRUCTURE analyses in the three categories, (a) all samples from the two species, (b) *A. takesimense*, and (c) *A. pseudosieboldianum*. We used an admixture model with correlated allele frequency (after this *F*-model; Falush et al. 2003) to assign individuals into *K* clusters (populations). 20,000 ‘‘burn-in’’ steps of Chain Monte Carlo searches, followed by 10,000 iterations, and 20 replicate runs were performed at each *K* from 1 to 10 under the *F*-model. We adopted the hierarchical approach for the STRUCTURE analysis employing ∆*K* to determine the uppermost level of structure (Evanno et al. 2005). In the *F*-model, *K* clusters are assumed to have diverged from a common ancestral population simultaneously, and the clusters may have experienced different degrees of genetic drift since the divergence event (Falush et al. 2003). Therefore, using the *F* model we can also estimate the amount of genetic drift in each of the different populations, estimated by *F* values.

To assess a recent population bottleneck, a graphical test to see whether the allele frequency distribution is approximately L-shaped or not (Luikart et al. 1998), and a test for the presence of an excess of observed heterozygosity by using the Wilcoxon signed rank test to evaluate departure from 1:1 deficiency/excess (Cornuet and Luikart 1996; Luikart et al. 1998) were conducted using BOTTLENECK 1.2.02 (Piry et al. 1999). In the latter test, heterozygosity excess was tested under the three mutation models, the infinite allele model (IAM; Kimura and Crow 1964), step-wise mutation model (SMM; Ota and Kimura 1973), and the two-phase model (TPM; Di Rienzo et al. 1994) with 1,000 simulation iterations. We set 90 % single-step, 10 % multiple-step mutations with a variance among multiple steps of 12 in the TPM.

## Results

### Genetic data analysis

Ten microsatellite loci were used to genotype 263 individuals from 14 populations in *Acer takesimense* and *A. pseudosieboldianum* (Table 1). All ten loci were amplified in all samples, but locus BDG7D showed complex patterns for genotyping in some samples. Hence, we used nine loci (excluding BDG7D) for further population analyses. The range of number of alleles in the nine loci was from five (A88LE and AVM1G) to 13 (A08Z1), and the mean was 8.6 in the two species. An exact test for HWE across populations and loci showed that 24 of 126 deviated from the HWE (*P* < 0.05) after Bonferroni correction. All the deviating cases were related to the positive *F*_IS_, indicating HWE deviation due to heterozygote deficit. Therefore, we estimated the frequency of null alleles across populations and loci using Micro-Checker, resulting in an average frequency from 0.228 (BDG7D) to 0.036 (A88LE), and 0.135 in all of the nine loci. For loci that showed significant heterozygote deficit, we generated corrected genotypic frequencies for putative null alleles using Micro-Checker. We performed population genetic analyses (AMOVA, pairwise genetic distance and bottleneck analyses) using both the corrected and non-corrected data sets. Significant linkage disequilibrium was not found between any pairwise loci in all populations (*P* < 0.05) after Bonferroni correction.

### Genetic diversity

Genetic diversity parameters estimated by the nine microsatellite loci are shown in Table 2. Populations of *A. takesimense* generally showed lower levels of genetic diversity than populations of *A. pseudosieboldianum* in allelic richness, expected heterozygosity, and total number of alleles according to the Mann–Whitney *U* test (*P* < 0.05). *F*_IS_ values were significantly positive in 11 populations, four of *A. takesimense* and all of *A. pseudosieboldianum* (*P* < 0.05) after Bonferroni correction. At the specific level, *A. takesimense* possessed half the number of private and rare alleles of *A. pseudosieboldianum*. If *A. takesimense* is treated as one population, then this population shows a large number of private and rare alleles in comparison with each of the seven populations of *A. pseudosieboldianum*.

**Table 2:** Genetic diversity parameters estimated by nine microsatellite loci in *Acer takesimense* and *A. pseudosieboldianum*

Taxon	Population	*A*_R_	*H*_O_	*H*_E_	*T*_A_	*F*_IS_	*N*_P_			*N*_R_		
							i	ii	iii	i	ii	iii
*Acer takesimense*							0.28	0.28		0.36	0.26	
	1	3.67	0.43	0.50	34	0.13			0.00			0.13
	2	4.23	0.33	0.57	39	**0.42**			0.00			0.06
	3	3.59	0.41	0.48	34	0.15			0.00			0.00
	4	3.83	0.30	0.55	35	**0.47**			0.04			0.11
	5	3.89	0.34	0.58	35	**0.41**			0.13			0.00
	6	3.85	0.47	0.51	36	0.08			0.00			0.03
	7	3.71	0.37	0.51	34	**0.27**			0.00			0.08
	Mean	3.82	0.38	0.53	35.3	**0.28**						
*A. pseudosieboldianum*							0.69			0.85		
	8	4.71	0.32	0.56	43	**0.43**		0.00	0.00		0.09	0.06
	9	4.91	0.46	0.67	45	**0.32**		0.00	0.00		0.05	0.05
	10	4.80	0.37	0.62	45	**0.41**		0.18	0.18		0.20	0.18
	11	4.02	0.41	0.56	37	**0.28**		0.00	0.00		0.05	0.00
	12	5.25	0.41	0.60	48	**0.32**		0.18	0.18		0.29	0.29
	13	3.79	0.42	0.53	35	**0.21**		0.03	0.03		0.03	0.03
	14	4.73	0.43	0.68	43	**0.37**		0.05	0.05		0.11	0.05
	Mean	4.60	0.40	0.61	42.3	**0.33**						

*A*_R_, allelic richness; *H*_O_, the observed proportion of heterozygotes, *H*_E_, the expected proportion of heterozygotes; *T*_A_, total number of alleles in nine microsatellite loci; *F*_IS_, the inbreeding coefficient (bold indicates departure significantly from zero); *N*_P_, the number of private alleles per individuals in each group; *N*_R_, the number of rare alleles per individuals in each group (defined as alleles with a frequency <10 % in the total group); The private and rare alleles were counted by (i) each species are treated as a group, (ii) the seven populations of *A. takesimense* as a group and the seven populations of *A. pseudosieboldianum* as seven different groups, (iii) each population in both species as a group

### Genetic structure

Genetic variation among and within populations was examined by AMOVA at different hierarchical levels with three categories using the corrected and non-corrected data sets (Table 3). The two data sets showed similar patterns; in all three categories, most of the variation was found among individuals within populations (79.0–96.2 %), and little genetic variation among populations (8.0 and 3.8 %) in the data sets of *A. takesimense*.

**Table 3:** Summary of analyses of molecular variance (AMOVA), showing degrees of freedom (*df*), sum of squares (SS), variance components, and the total variance contributed by each component (%) and its associated significance (*n* = 1,023 permutations)

Taxon	Source of variation	*df*	Non-corrected data set			Corrected data set		
			SS	Variance components	Total variance (%)	SS	Variance components	Total variance (%)
(a) *Acer takesimense* and *A. pseudosieboldianum*	Between taxa	1	108.1	0.366	11.7	35.5	0.128	16.6
	Among populations	12	142.3	0.249	7.9	22.6	0.034	4.4
	Within populations	512	1,293.2	2.526	80.4	311.3	0.608	79.0
(b) *A. takesimense*	Among populations	6	59.2	0.203	8.0	8.5	0.023	3.8
	Within populations	253	593.8	2.347	92.0	145.2	0.574	96.2
(c) *A. pseudosieboldianum*	Among populations	6	83.1	0.294	9.8	14.1	0.045	6.5
	Within populations	259	699.4	2.700	90.2	166.1	0.641	93.5

Results using both non-corrected and corrected data sets are shown

**Fig. 2:**
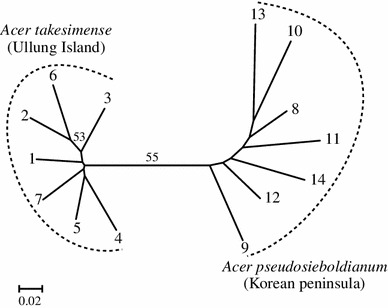
Neighbour-joining tree of the 14 populations of *Acer takesimense* and *A. pseudosieboldianum* based on *D*_A_ distance (Nei et al. 1983). Population numbers correspond to Table 1 and Fig. 1. Bootstrap probabilities >50 % are shown above the branches

A neighbour-joining tree of the two species was constructed based on *D*_A_ genetic distance (Fig. 2). The corrected and non-corrected data sets show much the same results; thus we present syntheses from the non-corrected data only. The populations of each species make two distinct clusters with 55 % bootstrap values. Genetic distances among populations of the same species were lower in *A. takesimense* (mean 0.105, SD 0.018) than in *A. pseudosieboldianum* (mean 0.152, SD 0.038) according to the Mann–Whitney *U* test (*P* < 0.05). The mean genetic distance between populations of the two species was 0.260 (SD 0.036).

**Fig. 3:**
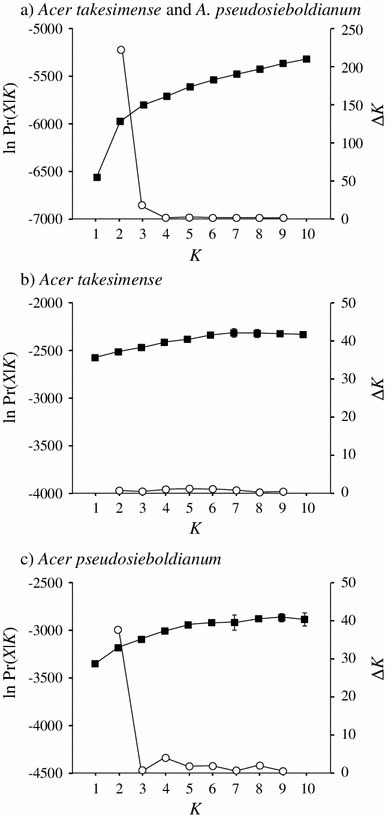
Results of Bayesian clustering (STRUCTURE, Pritchard et al. 2000) of *Acer takesimense* and *A. pseudosieboldianum*. **a** All the samples of *Acer takesimense* and *A. pseudosieboldianum*, **b** *A. takesimense*, and **c** *A. pseudosieboldianum*. The *solid square plots* give the mean ln Pr(*X*|*K*) and ±SD over 20 runs for each value of *K*. The *open circle plots* give Δ*K* of Evanno et al. (2005) showing a peak at the uppermost level of structure at the true value of *K*

**Fig. 4:**
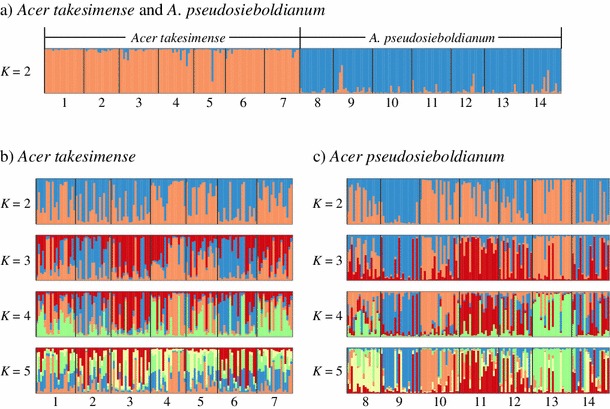
Results of Bayesian clustering (STRUCTURE, Pritchard et al. 2000) of *Acer takesimense* and *A. pseudosieboldianum*. *K* = 2 is shown in **a** all the samples of *Acer takesimense* and *A. pseudosieboldianum*, and *K* = 2, 3, 4 and 5 are shown in **b** *A. takesimense*, and **c** *A. pseudosieboldianum*. Each individual is represented by a *single vertical line* broken into *K* colored segment, with lengths proportional to each of the *K* inferred clusters. Population numbers below graph correspond to Table 1 and Fig. 1

The Bayesian clustering analyses implemented in STRUCTURE are shown in Figs. 3 and 4. The uppermost level of structure was at *K* = 2 based on Δ*K* value in two categories, (a) all samples from the two species, and (c) *A. pseudosieboldianum* (Fig. 3a, c). On the other hand, no obvious signal for the uppermost level of structure was detected in the category (b) *A. takesimense* (Fig. 3b). In all samples at *K* = 2, the two clusters correlated well with the two different species, because cluster I comprised 96 % of the genotypes in *A. takesimense* and cluster II comprised 94 % of the genotypes in *A. pseudosieboldianum* (Fig. 4a). The *F* value of cluster I (mean *F* = 0.194, SD 0.003) was higher than that of cluster II (mean *F* = 0.066, SD 0.002). In the separate analysis performed for each category, no clear populational subdivisions were found in each species (Fig. 4b, c).

### Population bottleneck

We tested bottleneck effects using the corrected and non-corrected data sets. They showed much the same results, and therefore again we dealt with results from the non-corrected data only. The mode-shift test detected the evidence of a bottleneck in Population 9 of *A. pseudosieboldianum* (Table 4). Excess heterozygosity was detected in Population 5 of *A. takesimense* and Populations 9 and 14 under IAM (*P* < 0.05) after Bonferroni correction. In Population 14 excess heterozygosity was detected under TPM as well. As a result, Populations 9 and 14 of *A. pseudosieboldianum* detected recent reduction of population size in multiple analyses (or mutation models).

**Table 4:** Summary of the parameters and results for BOTTLENECK analyses using non-corrected data sets

Taxon	Population	BOTTLENECK			
		Mode shift	IAM	TPM	SMM
*Acer takesimense*	1	NS	0.150	0.500	0.787
	2	NS	0.102	0.715	0.820
	3	NS	0.326	0.850	0.875
	4	NS	0.020	0.527	0.629
	5	NS	**0.001**	0.455	0.545
	6	NS	0.082	0.410	0.674
	7	NS	0.500	0.590	0.715
*A. pseudosieboldianum*	8	NS	0.367	0.936	0.993
	9	Shifted mode	**0.001**	0.082	0.285
	10	NS	0.064	0.545	0.787
	11	NS	0.082	0.367	0.455
	12	NS	0.213	0.787	0.936
	13	NS	0.019	0.285	0.590
	14	NS	**0.001**	**0.002**	0.007

*NS* no significance (*P* < 0.05), *bold* significant (*P* < 0.05) after Bonferroni correction

## Discussion

### Genetic differentiation between the two species

The genetic difference between the endemic Ullung Island species *Acer takesimense* and its progenitor *A. pseudosieboldianum* was clear in both the STRUCTURE analyses and neighbour joining tree. In the STRUCTURE analyses, most individuals were assigned to each of the species clusters, but there were a few individuals that had intermediate genetic components between the two species (Fig. 4a). Although this might be explained by interspecific gene flow, a more plausible explanation might be limited statistical power or size homoplasy of microsatellites. Due to the isolated geographical distribution of the two species, restricted to the oceanic island or continental region, the opportunity for interspecific gene flow would be rare. Previous AFLP analyses showed consistent patterns for clear genetic differences between the two species and *trnL*–*F* sequences of cpDNA also had one nucleotide substitution between the two species (Pfosser et al. 2002). No difference has been found in ITS sequences, however, between the two (Cho et al. 1996).

The two species differ in morphological features, with *A. takesimense* having deeply divided smaller leaves with usually 13 or more lobes (Sun and Stuessy 1998), and *A. pseudosieboldianum* having larger leaves that are much less divided (Park et al. 1993, 1994; Sun and Stuessy 1998). *Acer takesimense* is also typically more strongly branched than *A. pseudosieboldianum* (van Gelderen et al. 1994). Despite these differences, their taxonomic status has been re-evaluated by Chang (1994), concluding that the two species should be merged into one. Acknowledging that the two taxa are closely related, it is our view that the results support specific recognition based on the following arguments: (1) each represents a single cluster, with the average of population differentiation between the two (*D*_A_ = 0.260) being much higher than that among populations of *A. pseudosieboldianum* (*D*_A_ = 0.152); (2) the populations of *A. takesimense* accumulate some private and rare alleles, suggesting genetic uniqueness; and (3) the two populational systems are completely geographically isolated.

### Genetic diversity within species and populations

Genetic diversity in terms of allelic richness, expected heterozygosity, and total number of alleles is generally lower in the populations of *Acer takesimense* in comparison with those of *A. pseudosieboldianum*. A smaller number of alleles in populations of *A. takesimense* was also revealed by AFLP analysis (Pfosser et al. 2002). A reduction of genetic diversity in island populations has been documented in many instances (Frankham 1997). For endemic island plants, low levels of genetic diversity have been commonly observed in Hawaiian, Galapagos, Juan Fernandez and Bonin Islands (Crawford et al. 2001, 2006; DeJoode and Wendel 1992; Ito et al. 1998). A number of factors may be responsible for explaining the low levels of genetic diversity seen in endemic island populations, such as a bottleneck effect during immigration if it were of relatively recent origin, small population sizes, loss of populations and genes through island subsidence and erosion, high levels of inbreeding, and cladogenetic speciation. Another possibility is ascertainment bias of the microsatellite markers. The microsatellite markers used in this study were isolated from *A. pseudosieboldianum* (Takayama et al. 2011). The source species used in isolation of microsatellite markers is sometimes observed to be more polymorphic than the other species (Ellegren et al. 1997; Forbes et al. 1995).

In Ullung Island, another endemic *Acer* species, *Acer okamotoanum*, also showed slightly lower levels of genetic diversity in comparison with its progenitor species in the Korean Peninsula and Japan (Takayama et al. 2012). In both endemic species of *Acer*, recent population size reductions were not clearly detected by statistical tests for bottlenecks based on the allelic distribution, but strong episodes of genetic drift during colonization and speciation on the island were detected, because the *F* value in STRUCTURE inferred for these island species was quite high. Estimated population sizes of these island endemics were generally smaller than those of the continental progenitors. Reasons for the slightly lower levels of genetic diversity in the two endemic *Acer* species in Ullung Island, therefore, might be due to a combination of bottleneck effects resulting from immigration, young island age (1.8 million years, Kim 1985), and small population sizes.

A rather different genetic pattern has been documented in *Dystaenia takesimana* (Apiaceae), another endemic species in Ullung Island, which showed a slightly higher level of genetic diversity of AFLPs than its continental progenitor *D. ibukiensis* in the Japanese archipelago (Pfosser et al. 2005). Pfosser et al. (2005) concluded that *D. takesimana* may have regained genetic diversity during or after speciation along with increasing sizes of populations, because the estimated sizes of populations analysed were similar or larger than those of *D. ibukiensis* in the Japanese archipelago. The equally large sizes of the island populations of *D. takesimana* could be an important factor for explaining their high level of genetic diversity. The differences of genetic patterns between endemic species of *Acer* and *Dystaenia* may also relate to their distinct reproductive features. *Acer takesimense* and *A. okamotoanum* are trees that take several years for flowering (most *Acer* species first flower at 5–20 years, van Gelderen et al. 1994), but *Dystaenia takesimana* is a perennial herb that flowers annually. In addition, *Acer* species often are self-compatible (Gleiser and Verdú 2005; Gleiser et al. 2008; Kikuchi et al. 2009), but *D. takesimana* shows characteristics of xenogamous plants based on pollen-ovule ratios and AFLP analysis (Pfosser et al. 2005). The positive *F*_IS_ found in some populations of *A. takesimense* and *A. pseudosieboldianum* also support possible inbreeding in these species.

### Geographical genetic structure within Ullung Island and the Korean Peninsula

During anagenetic speciation, an initial founder lineage simply changes through time without further specific differentiation (Stuessy 2007; Stuessy et al. 2006). It is reasonable to assume, therefore, that anagenetically derived species could accumulate genetic variation through time without any eco-geographical partitioning of this variation. *Acer takesimense* possesses half the number of private and rare alleles of *A. pseudosieboldianum*, but considering the difference of total distribution of the two species (small oceanic island vs. Eastern Asia), the number of alleles in *A. takesimense* is relatively high. Over generations, genetic variation in *A. takesimense* may indeed have accumulated through mutation and recombination followed by changes in allelic frequencies through drift and/or selection. The seven populations of *A. takesimense* analysed were collected broadly at different elevations (120–900 m) on Ullung Island, but only weak geographical genetic structure was detected in STRUCTURE analysis. The level of genetic variation among populations was also quite low as documented with AMOVA. Such a weak geographical genetic structure within Ullung Island has also been reported in the anagenetically derived species, *A. okamotoanum* and *Dystaenia takesimana* (Pfosser et al. 2002; Takayama et al. 2012). These results are consistent, therefore, with our initial hypothesis on the genetic consequences of anagenetically derived species. It is also clear that not all species originating by this mode will have responded genetically in exactly the same way, depending upon their history and biology. One might also wish to argue that the results have been caused by low resolution of molecular markers. We consider that the markers used in this study are high enough to detect polymorphisms among individuals, because the average expected heterozygote of nine nuclear microsatellite markers was 0.53 and the probability of identity of multilocus genotypes using the nine markers was extremely low (0.0013 %). The weak geographical genetic structure, therefore, suggests that genetic exchange among populations of *A. takesimense* may be occurring frequently throughout Ullung Island.

*Acer pseudosieboldianum* from continental regions, on the other hand, shows a noticeable genetic differentiation in STRUCTURE analysis, because a clear signal was found at *K* = 2 based on Δ*K* value. The stronger genetic differentiation found in *A. pseudosieboldianum* might be explained by the larger number of individuals and its broader total distribution. We used seven populations of *A. pseudosieboldianum* widely distributed from the southern part of the Korean Peninsula. Since the biological features such as generation time, habitat preference, and mode of seed and pollen dispersal would be similar in the two species, wider distribution of *A. pseudosieboldianum* easily allows the differentiation of populations through fixation of alternative alleles, local adaptation, and genetic drift involved with demographic changes.

In the structure analysis, the compositions of clusters between Populations 9 and 13 were quite different from each other, but there were also multiple clusters mixed together within the other populations. It is difficult, therefore, to see any trend in geographic structure due to genetic variation. This complex geographic structure could be the result of their ability for long distance dispersal of seeds (via the well-known winged samara fruits) and subsequent genetic exchange, and also changes in population sizes and distribution throughout the Quaternary glacial period. Macrofossils and pollen data from the Korean Peninsula indicate that temperate deciduous broad-leaved forests broadly declined between ca. 20,700 and 11,500 years BP, and increased after ca. 11,500 years BP in response to dramatic climate change during the late Pleistocene and early Holocene (Chung et al. 2010; Kong 2000). The genus *Acer* is a one of the principal components of the deciduous broad-leaved forests in this region, and they would have undergone dynamic historical changes in population size and distribution in the Korean Peninsula. Although our molecular samples at the populational level were not collected for the purpose of completing a detailed phylogeographic analysis of the Korean Peninsula, severe recent (i.e., maximum 4*N*e generations ago) bottleneck signals were detected in the two populations (9 and 14) of *A. pseudosieboldianum*, which could support the concept of recent dramatic distributional changes within this species. Further study will be required to provide a foundation for phylogeographic evolutionary insight into progenitor species in the Peninsula.

This study adds important new data on the genetic consequences of anagenetic speciation in oceanic islands. In cladogenesis, several lines of speciation occur from founder populations by selection within markedly different ecological zones that results in different morphological and physiological traits, and also in genetic portioning of the founder populations. In anagenetic speciation, as shown in this study, after a founder event the initial population expands its distribution range across the entire island, and in the process accumulates genetic variation through mutation and recombination. No eco-geographic partitioning of genetic variation results. Over many generations, island populations simply diverge in genetic and morphological composition from progenitor populations, and a new species arises.
